# Materials Formed by Combining Inorganic Glasses and Metal‐Organic Frameworks

**DOI:** 10.1002/chem.202200345

**Published:** 2022-05-25

**Authors:** Ashleigh M. Chester, Celia Castillo‐Blas, Lothar Wondraczek, David A. Keen, Thomas D. Bennett

**Affiliations:** ^1^ Department of Materials Science and Metallurgy University of Cambridge 27 Charles Babbage Road CB3 0FS Cambridge UK; ^2^ Otto Schott Institute Materials Research University of Jena Fraunhoferstrasse 6 07743 Jena Germany; ^3^ ISIS Facility Rutherford Appleton Laboratory Harwell Campus OX11, 0DE, Didcot Oxfordshire UK

**Keywords:** hybrid composite, hybrid glass, metal-organic framework, new materials

## Abstract

Here, we propose the combination of glassy or crystalline metal‐organic frameworks (MOFs) with inorganic glasses to create novel hybrid composites and blends.The motivation behind this new composite approach is to improve the processability issues and mechanical performance of MOFs, whilst maintaining their ubiquitous properties. Herein, the precepts of successful composite formation and pairing of MOF and glass MOFs with inorganic glasses are presented. Focus is also given to the synthetic routes to such materials and the challenges anticipated in both their production and characterisation. Depending on their chemical nature, materials are classified as crystalline MOF‐glass composites and blends. Additionally, the potential properties and applications of these two classes of materials are considered, the key aim being the retention of beneficial properties of both components, whilst circumventing their respective drawbacks.

## Graphical abstract

**The formation of novel hybrid composites** through the combination of the chemically versatile and functional metal‐organic framework (MOF) family, with ubiquitous, low cost and durable inorganic glasses is explored in this Concept article. The blends and composites created from this combination may serve to minimise, and eventually solve cost, durability and processability issues of MOFs, whilst retaining their valuable properties.

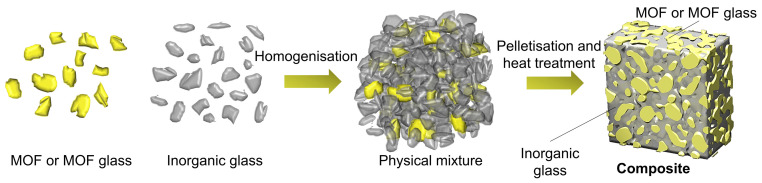

## Introduction

Over the last two decades, metal‐organic frameworks (MOFs) have had the attention of materials‐focused researchers on account of their intrinsically tuneable chemical structures, flexible architectures, high surface areas and multifunctional properties. These innovative materials offer the potential for use in multiple high‐value applications such as catalysis,^[1]^ controlled drug delivery,^[2]^ and gas storage or separation.^[3]^

MOFs are defined as hybrid crystalline networks constructed via the self‐assembly of an inorganic metal cluster and an organic linker, resulting in a three‐dimensional periodic porous material.^[4]^ To date, MOFs are conventionally synthesised as microcrystalline powders, which creates practical and commercial barriers caused by their poor processability and weak mechanical performance.^[5]^ To address this, the idea of synthesising bulk materials through pelletisation or monolith preparation using sol‐gel techniques has evolved.^[6]^ However, the efficacy of the MOF material is often compromised, specifically with respect to its porosity and functionality.^[7]^ As such, interest in combining MOFs with more processable materials to form new composites has expanded in recent years. Examples of the composite approach range from mixed‐matrix membrane systems (MMMs) to MOF‐in‐silica core‐shell structures.^[8]^ These approaches offer solutions to manufacturing problems, and sometimes reduce the total material cost. However, given the issues surrounding current MOF composites, our goal is to explore the concept of combining a MOF with an inorganic glass matrix.

Inorganic glasses are amorphous materials^[9]^ which exhibit a transition from a brittle solid to a viscoelastic state, characterised by the glass transition temperature (*T*_g_) and the viscosity gradient at this temperature (liquid fragility index, *m*). Transition into the glassy phase occurs over a temperature range around *T*_g_, where liquid disorder is frozen‐in.^[10]^ Classically, production of the glass phase involves melt‐quenching, where the liquid is cooled sufficiently fast to avoid molecular or atomic reorganisation i. e. crystallisation. While in principle any liquid can be transformed into a glass, glasses are commonly classified according to their chemical composition, namely inorganic (silicate, oxide, non‐oxide), organic, metallic, and hybrid glasses such as MOF and coordination polymer (CP) glasses.

Currently, inorganic glasses are ubiquitous in applications ranging from containers and architecture to optical fibres, electronic packaging and display screens.^[11]^ Ways to address the increasing demand for thin and flexible glasses could include toughening strategies and composite formation with other materials.^[12]^ Combining them with MOF glasses or crystalline MOFs may result in materials which exhibit the advantages of both MOF and inorganic glass phases.

**Figure 1:**
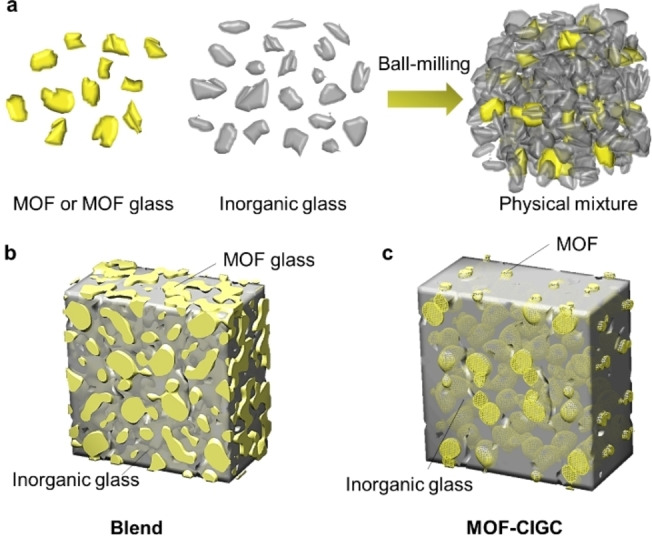
**a**. Homogenous mixing of an inorganic glass and MOF component via ball‐milling prior to heat treatment, **b**. MOF‐CIGC (Metal‐Organic Framework Crystal Inorganic Glass Composite) and **c**. Homogenous blend of a MOF glass and inorganic glass.

We classify such materials into two categories: blends and composites (Figure 1). A blend can be considered a macroscopically homogeneous mixture of two or more different materials that both enter their low viscosity regime during blend formation. By allowing both components to access their liquid phase during heating, a translucent and amorphous blend can be formed after quenching. In contrast, MOF composites involve a similar synthesis method, but the MOF particles remain solid while the glass enters its liquid phase and flows around the particles. A non‐translucent material is produced and X‐ray diffraction patterns from the composite will contain Bragg peaks corresponding to the unaltered MOF structure. Such a composite can be defined as a “multicomponent material comprising multiple, different (non‐gaseous) phase domains”.^[13]^

## Initial Considerations

**Figure 2:**
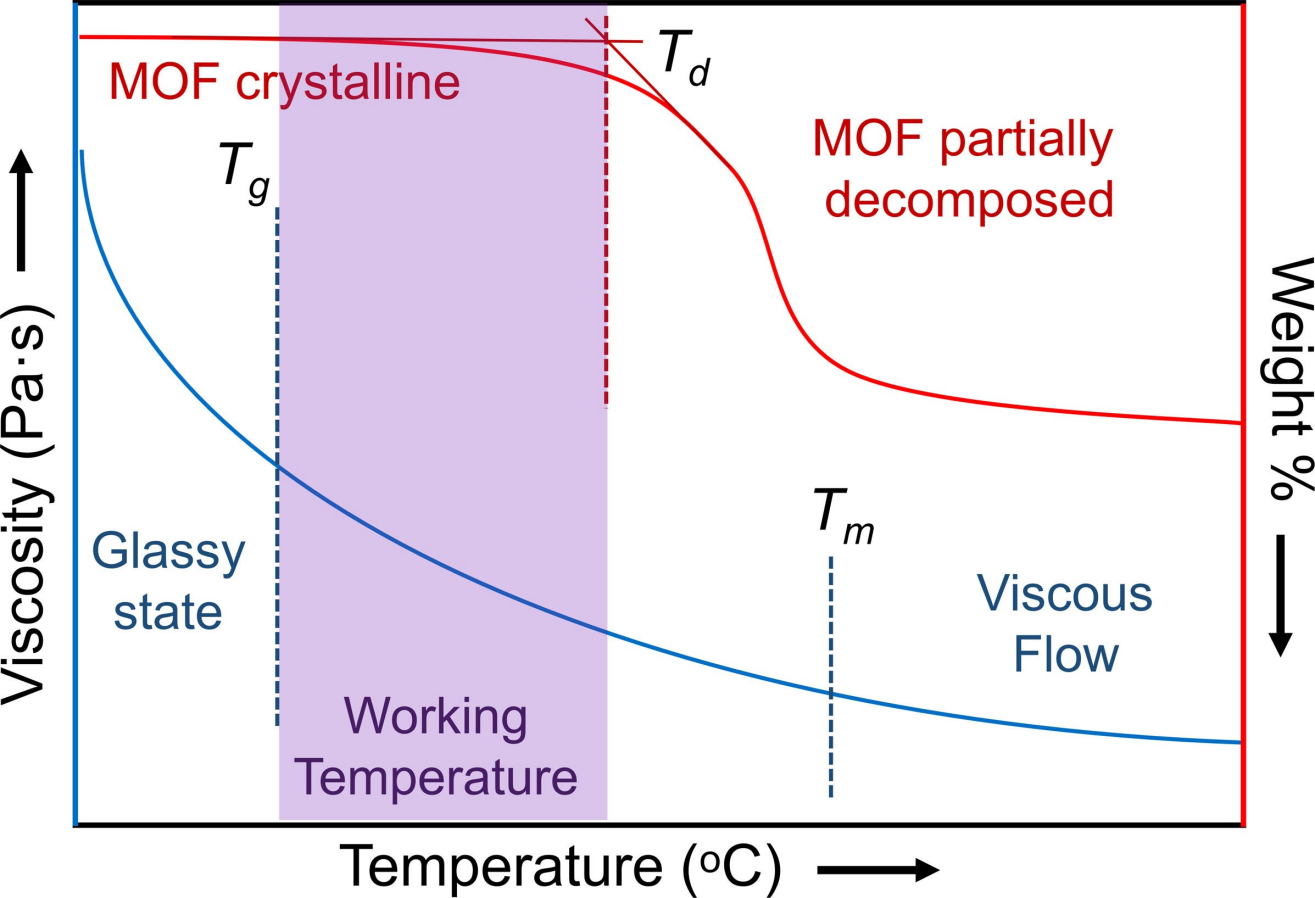
Viscosity vs. Temperature of an inorganic glass (blue) and thermalgravimetric analysis of a metal‐organic framework (red). Working temperature should have a value between *T*_g_ of the glass and *T*_d_ of the MOF.

For successful blend and composite formation by thermal treatment (e. g. co‐melting, co‐sintering), two criteria are important: thermal and chemical compatibility. Thermal compatibility arises when the glass transition temperature of the glass is sufficiently below the decomposition temperature (*T*_d_) of the MOF, to prevent degradation of the MOF component. The working temperature (*T*_w_) is the maximum temperature (usually dwell temperature) used during the thermal treatment of the composite or blend and must be between *T*_g_ and *T*_d_. This is crucial because the *T*_w_ needs to exceed the *T*_g_ of the glass to decrease the viscosity of the liquid enough to promote effective mixing with the MOF particles (Figure 2). Examples of different inorganic glasses are shown in Table 1.

**Table 1:** Examples of various inorganic glasses and their glass transition temperatures.

Inorganic glass	*T*_g_ [°C]	Ref.
xAgI(1‐x)AgPO_3_ (x=0–0.5 mol%)	80–186	^[14]^
(100‐x)NaPO_3_‐xAlF_3_ (x=0–30 mol %)	289–367	^[15]^
xZnO‐(1‐x)TeO_2_ (x=0–0.45 mol%)	303–352	^[16]^
45Na_2_O‐xAl_2_O_3_(55‐x)‐P_2_O_5_ (x=3–10 mol%)	334–443	^[17]^
(25‐x)K_2_O‐xLi_2_O−25Al_2_O_3–_50B_2_O_3_ (x=0–25 mol%)	400–449	^[18]^
45SiO_2–_24.5Na_2_O−24.5CaO−6P_2_O_5_ (bioglass)	550	^[19]^

Given the low *T*_d_ values of most MOFs (*T*_d_<350 °C) and their glasses, only glasses with *T*_g_s lower than classical SiO_2_‐based glasses (ranging from about 530 °C for window glass to *T*_g_ > 1200 °C for vitreous silica) are suitable for pairing with MOFs.^[20]^ Examples of such low *T*_g_ glasses include borate, fluorozirconate, or phosphate‐based inorganic glasses, the *T*_g_ values of which can be adjusted by the addition of modifier oxides and fluorides such as Na_2_O, Bi_2_O_3_, LaF_3_ or AlF_3_.^[21]^ However, many other candidate formulations for low ‐melting glasses, for example those comprising bismuthates or lead oxide,^[22]^ are not suitable because of their chemical reactivity with the organic linker of the MOF.

Other available glass matrix candidates are hybrid glasses, such as the recently discovered MOF glasses. Several MOFs have been identified to exhibit a stable liquid phase after heating above the melting temperature (*T*_m_), which can form a glass upon subsequent cooling. The ability to form a liquid phase is thought to increase the workability of the material relative to its crystalline analogue as the MOF liquid can be moulded into various shapes and/or blended with other materials.^[23]^

The MOF glasses that have been synthesised thus far have been limited mainly to zeolitic imidazolate frameworks (ZIF), which consist of tetrahedrally‐coordinated metal ions (e. g. Zn^2+^, Co^2+^) and imidazolate‐derived linkers.^[24]^ In addition to ZIFs, several other coordination polymer (CP) and crystalline MOF families have exhibited glass forming ability (Table 2).^[25]^

**Table 2:** Examples of several glass‐forming MOFs and CPs.

MOF/CP	*T*_m_ [ °C]	*T*_g_ [ °C]	Ref.
**ZIF‐62**: Zn(Im)_2–*x*_(bIm)_*x*_ (*x*=0.05–0.35)	372–441	298–320	[26–28]
**ZIF‐62 (Co)**: Co(Im)_2–*x*_(bIm)_x_ (*x*=0.10–0.30)	386–432	260–290	[26,27]
**ZIF‐UC‐2**: Zn(Im)_1.90_(6‐Cl‐5‐FbIm)_0.10_	406	250	[29]
**ZIF‐UC‐3**: Zn(Im)_1.75_(5‐Cl‐2‐mbIm)_0.25_	390	336	[29]
**ZIF‐UC‐4**: Zn(Im)_1.63_(5‐FbIm)_0.37_	421	290	[29]
**ZIF‐UC‐5**: Zn(Im)_1.69_(5‐ClbIm)_0.31_	432	320	[29]
**TIF‐4**: Zn(Im)_1.5_(mbIm)_0.5_	440	350	[27]
**[Ag(*p*L2)(CF_3_SO_3_)]** ⋅ **2 C_6_H_6_**	271	161	[30]
**[Ag(**m**L1)(CF_3_SO_3_)]** ⋅ **2 C_6_H_6_**	169	68	[30]
**[Cu_2_(SCN)_3_(C2bpy)] [Cu_2_(SCN)_3_(C4bpy)] [Cu_8_(SCN)_12_(Phbpy)_4_] [Cu(SCN)_2_(3‐Pybpy)]**	187 138 217 203	68 59 71 72	[31] [31] [31] [31]

Ligand abbreviations. Im=imidazolate, bIm=benzimidazolate, 6‐Cl‐5‐FbIm=6‐Chloro‐5‐fluorobenzimidazolate, 5‐Cl‐2‐mbIm=5‐Chloro‐2‐methylbenzimidazolate, 5‐FbIm: 5‐Fluorobenzimidazolate, 5‐ClbIm: 5‐Chlorobenzimidazolate, mIm: 2‐methylimidazolate, *p*L2=1,3,5‐tris(4‐ethynylbenzonitrile)benzene, *m*L1=1,3,5‐ tris(3‐cyanophenylethynyl)benzene), C2bpy=1‐ethyl‐ [4,4’‐bipyridin]‐1‐ium, C4bpy=1‐butyl‐[4,4’‐bipyridin]‐1‐ium, Phbpy=1‐ phenyl‐[4,4’‐bipyridin]‐1‐ium, 3‐Pybpy=[3,1’:4’,4”‐terpyridin]‐1’‐ium.

Given the extensive range of *T_g_* s of inorganic glasses, which can be modified by tailoring glass composition (Table 1), there is a plethora of inorganic glasses available for blending with hybrid glasses. As such, *T_g_* values of both can be closely matched, however this is not a prerequisite for successful blend formation. As long as the liquid phases of both the inorganic glass and hybrid glass are stable at the *T_w_* selected, no decomposition of either starting material should theoretically occur. Moreover, a meltable hybrid glass former can be heated in its crystalline phase with an inorganic glass if the latter's *T_g_* is too high. This is because the *T_m_* of the crystalline phase is usually higher than the *T_g_* of the glass that is formed after melt‐quenching.

The other important consideration is chemical compatibility, which refers not only to the redox potential of the composite partners, but also to the bonding compatibility between the glass matrix and the MOF. The creation of a robust material is realised through effective bond formation at the interface between the individual constituents of the composite. Such interfacial bonding results in properties which are specific to the composite beyond simple component mixing. For example, non‐linear variations in ionic mobility or mechanical behaviour have been reported for blends combining inorganic glasses and glass‐MOF materials.^[32]^

However, evaluating chemical compatibility is a major challenge, but insights into bonding interactions can be provided by nuclear magnetic resonance, infrared and Raman spectroscopy.^[32]^ Additionally, a homogenous mixture of both components can be achieved through efficient mixing prior to heat treatment via two potential different routes. One of these routes may include the in situ nucleation of the MOF on top of the inorganic glass while the other involves mixture homogenisation via ball‐milling and pelletisation. However, loss of crystallinity of MOFs has been reported after excessive ball‐milling and pressure applied, which can be avoided by adjusting these parameters (time, frequency applied and pressure).^[33]^ With these considerations, suitable MOF and inorganic glass components can be paired.

## Classification of MOF‐Glass Composites

### MOF glass‐inorganic glass blends

Blending components to form new, homogenous materials is the basis of traditional glass chemistry, where properties are tuned over wide ranges by mixing various chemical compounds.[^34^, ^35^] Similarly, polymer blending is applied in the plastics industry to create new functional materials, and hybrid polymers formed by blending the melts of hydrocarbons and inorganic compounds are representative of a new class of polymers. Typical inorganic polymers include polyoxides, borides, phosphides and monoelement polymers (e. g. C_n_, Si_n_, Te_n_, Se_n_, B_n_) which can be blended with conventional organic polymers.^[36]^ One such example was a blend created from boron polyoxides (BPOs) and low‐density polyethene (LDPE). The planarity of the BPO polymer was thought to decrease the viscosity in the melt flow, creating a blend with increased elastic modulus and tensile strength. Additionally, the strength of the blends was shown to increase with an increasing proportion of the inorganic constituent.

More recently, attempts to blend MOF liquids ZIF‐4‐Co, [Co(Im)_2_]_,_ Im: imidazolate (C_3_H_3_N_2_^−^) and ZIF‐62, [(Zn(Im)_1.75_(bIm)_0.25_], bIm: benzimidazolate (C_7_H_5_N_2_^−^) were made, in which the liquid phase mixing of the MOF components produced a glass that had a single *T*_g_ and exhibited heterogenous domain locking.^[37]^ Liquid phase mixing of MOFs can be achieved by heating to temperatures exceeding the *T*_m_s of both components. Following heat treatment, cooling to room temperature can create a blend with interlocking MOF domains. Alternatively, flux melting can be achieved when a liquid MOF is used to facilitate melting of a different MOF with an inaccessible *T*_m_, as demonstrated by a flux‐melted glass, a_g_[(ZIF‐67)_0.2_(ZIF‐62)_0.8_] where a_g_ stands for ‘amorphous phase formed via melt‐quenching’ and ZIF‐67 is [Co(mIm)_2_], mIm: 2‐methylimidazolate.

**Figure 3:**
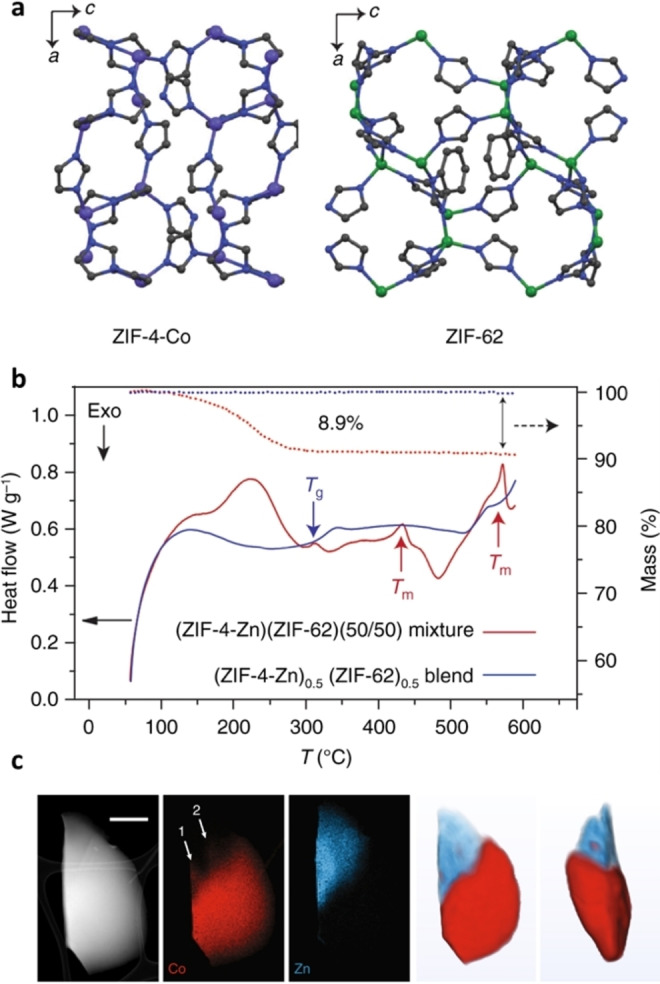
**a**. unit cell of ZIF‐4‐Co and ZIF‐62, both viewed down their crystallographic *b* axes; **b**. DSC scan of the physical mixture (ZIF‐4‐Zn)(ZIF‐62)(50/50) and corresponding blend; **c**. Two‐dimensional analysis by ADF‐STEM showing the particle morphology and EDS chemical maps of Co and Zn. Scale bar is 500 nm; **d**. A volume rendering of the tomographic reconstructions for the Co and Zn signals (two orthogonal viewing directions). Dark blue, grey, green and purple represent nitrogen, carbon, zinc and cobalt respectively. Hydrogen atoms have been omitted (Copyright 2018, Nature Comm.).^[38]^

This approach was also used for the blending of ZIF‐4‐Zn and ZIF‐62, where sufficient intra‐domain connectivity was observed when the mixture was heated at a constant temperature above the *T*_m_s of both components (Figure 3).^[38]^ Upon re‐heating, a single *T*_g_ at 306 °C was observed, instead of the expected *T*_g_ features of 292 °C (ZIF‐4‐Zn) and 318 °C (ZIF‐62), which confirmed liquid phase mixing. The successful mixing was attributed to chemical compatibility between the ZIFs, but complete homogeneous mixing could not be achieved because of the high viscosity of the liquid phases.

Blending inorganic glasses with organic polymers is another avenue to new composites with different properties. Common examples include blending phosphate glasses with polymeric materials such as LDPE, polypropylene, polystyrene and polyphenylene sulfide.^[39]^ Organic polymers typically have lower *T*_g_s than inorganic glasses and thus it is beneficial to blend polymers with low *T*_g_ glasses (∼200–350 °C). For example, blending zinc alkali phosphate glasses with polymers combined the stiffness and strength of the inorganic glass with the light weight and processability of the organic polymer, with potential applications in aerospace, automotive and electrical industries.^[40]^

Building on this concept, recent work focused on the blending of a_g_ZIF‐62 and an inorganic glass series (1–x)([Na_2_O]_z_[P_2_O_5_])‐x([AlO_3/2_][AlF_3_]_y_). Here, the formation of interlocked a_g_ZIF‐62 and inorganic glass domains was observed after melt‐quenching at a temperature exceeding both the inorganic glass *T*_g_ and the *T*_m_ of ZIF‐62.^[32]^ Chemical mixing and interfacial bonding between the domains was confirmed by EDX spectroscopy and solid‐state NMR, respectively. The composites were found to be more mechanically pliant than the parent inorganic glass, but instead of a homogenous blend, a heterogenous composite with two *T*_g_s was produced. This work provided the first example of combining a MOF glass with an inorganic glass and serves as a foundation of MOF glass‐inorganic glass blend research.

### MOF crystal‐inorganic glass composites

A metal‐organic framework crystal‐inorganic glass composite (MOF‐CIGCs) is synthesised via the dispersal of a crystalline MOF within an inorganic glass matrix at a working temperature where the latter can flow around the embedded MOF particles. This approach is based on previous work on similar composites in which MOF particles were embedded in a MOF (hybrid) glass matrix; these are designated MOF crystal‐hybrid glass composites (MOF‐CHGC).^[41]^

A recent example of a MOF‐CIGC involved the combination of a sodium fluoroaluminophosphate glass series, 78 %([Na_2_O]_1.6_[P_2_O_5_])–22 %([AlO_1.5_][AlF_3_]_0.7_) with ZIF‐8 [Zn(mIm)_2_]. Suitable thermal compatibility was rationalised, given that the *T*_g_ of the glass (352 °C) was much lower than the *T*_d_ for ZIF‐8 (520 °C).^[42]^ Varying amounts of ZIF‐8 were used and 450 °C was selected as the working temperature because it was between the inorganic glass *T*_g_ and the *T*_d_ of ZIF‐8. However, analysis on the synthesised composites suggested that although some interaction had occurred between the inorganic glass and ZIF‐8, this resulted in partial destruction of the ZIF‐8 component. This was evidenced through mass loss of the crystalline component, and highlighted the challenges associated with finding the right inorganic glass and MOF pair.

Given these decomposition considerations, the selection of an inorganic glass with a lower *T*_g_ is essential for MOF‐CIGC formation. Examples of low *T*_g_ glasses include the glass series (Na_2_O)_x_(P_2_O_5_)_100‐x_ (10≤x≤50)^[43]^ and fluorozirconate glasses, such as ZBLAN (ZrF_4_−BaF_2_−LaF_3_−AlF_3_−NaF), which both have *T*_g_s lower than 300 °C.^[44]^ These are potential candidates for the glass matrix and can be paired with MOFs which are stable at temperatures up to 300 °C.

Options for the crystalline MOF component include rigid MOFs such as UiO‐66, ZIF‐8, ZIF‐67 and MIL‐125, or MOFs with a degree of flexibility, such as DUT‐49, MIL‐88 or MIL‐53, which exhibit different conformations. One of the advantages of combining flexible MOFs with an inorganic glass matrix could be the immobilisation of a higher surface area conformation within the composite.[^45^, ^46^] This concept has been demonstrated by a MOF‐CHGC in which the composite comprised ZIF‐62 glass and a stimuli‐responsive MOF, MIL‐53(Al).^[47]^ The as‐synthesised structure MIL‐53 (MIL‐53‐as) contains guest molecules within its pores that can be driven off by heating, resulting in an open pore, metastable form, MIL‐53 (lp) where lp=large pore. However, MIL‐53(lp) reverts to the narrow pore conformation, MIL‐53 (np), at room temperature. Interestingly, combining MIL‐53 with a glass matrix stabilised the large pore conformation, in which the coordinative bonding and chemical structure of MIL‐53 was preserved within the matrix. Moreover, the presence of nanoscale interfacial interaction led to improved mechanical properties of the composite relative to the starting materials.^[41]^

A similar approach was taken to synthesise MOF‐CHGCs with other MOFs and a_g_ZIF‐62 at the same working temperature (450 °C), these included UiO‐66, ZIF‐67, Cumof‐9, DUT‐6, DUT‐8, MIL‐68, MIL‐118, MIL‐120, MIL‐126(Sc) and UL‐MOF‐1.[^48^, ^49^] However, the majority of the MOFs exhibited thermal decomposition during composite formation because of the high working temperature used, which was originally selected as it was sufficiently higher than the melting temperature (*T*_m_) of ZIF‐62. These attempts accentuated the need for adequate thermal and chemical compatibility of the individual composite components.

### Potential Applications

Given the functional diversity of the precursor materials, potential applications of these novel materials are extensive because the MOF and glass properties can be combined or improved in the composite material. More generally, creating composites may significantly reduce the time and economic constraints associated with synthesising new materials from scratch. The economic cost of MOFs and MOF glasses in particular would be circumvented by combining them with cheaper, more processable materials such as inorganic glasses.

More specifically, use of composites for storage and gas separation may be a particularly promising application. As a proof of concept, this has already been demonstrated by a MOF‐CHGC where stabilisation of the porous conformation, MIL‐53(lp), led to significant improvement of CO_2_ sorption at room temperature, exceeding that of the precursors.^[47]^

**Figure 4:**
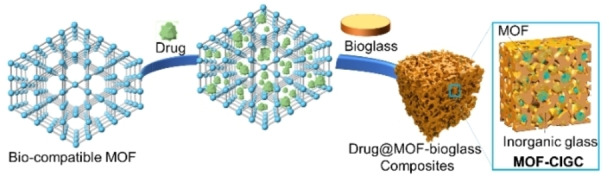
Schematic representing the encapsulation of a drug in a biocompatible MOF that is combined with a bioglass to form a MOF‐CIGC.

Additionally, the use of bioglasses, such as (Na_2_O)_x_(P_2_O_5_)_100‐x_, with biocompatible MOFs, for example MIL‐100, could have biomedical applications. Crystalline MOFs have already been proposed for use in drug delivery systems; examples include alleviating bacteriological risk and delivering chemotherapy drugs.^[2]^ Combining these MOFs with a bioglass matrix could mitigate the processability issues associated with MOFs (Figure 4) while at the same time incorporating a biocompatible drug delivery system. For implants specifically, calcium‐based bioactive MOFs might be promising candidates to pair with bioglasses because they may promote osteostimulation by generating hydroxyapatite.^[50]^

Another potential application is the use of photoactive MOFs such as MIL‐125 for self‐cleaning windows, in which combining a MOF with a more processable material would be beneficial. Under UV‐VIS radiation, particles of the MOF within the composite might first capture, and subsequently facilitate the catalytic degradation of pollutants and toxic chemicals.^[51]^

Furthermore, the ability to selectively uptake water is now becoming known as a quintessential property of the MOF family, such as MOF‐808, MIL‐101 and others. The combination of these MOFs with hygroscopic inorganic glasses may well improve the amount of water uptake in areas of relatively low humidity. This idea may also be extended to blending hygroscopic inorganic glasses with ZIF glasses for proton conductivity applications to compete with Nafion, a material that requires high relative humidity conditions.^[52]^

### Characterisation and Challenges

The nature of the selected glasses gives rise to important storage considerations; hygroscopic glasses such as those based on B_2_O_3_ and P_2_O_5_ network formers require storage in a dry environment, as would any blends and composites that incorporate them. Characterisation of these composites and blends, should involve multiple techniques. Powder X‐ray diffraction (PXRD) is critical for confirming phase purity of the crystalline MOF in MOF‐CIGCs, and to confirm the maintenance of the amorphous nature of the glass after thermal treatment in blends and MOF‐CIGCs. Thermal gravimetric analysis (TGA) and PXRD are essential for confirming that no decomposition or recrystallisation of either starting material occurs during heat treatment. Differential scanning calorimetry (DSC) is also crucial for obtaining the *T*_g_s of the resulting blend/composite.

**Figure 5:**
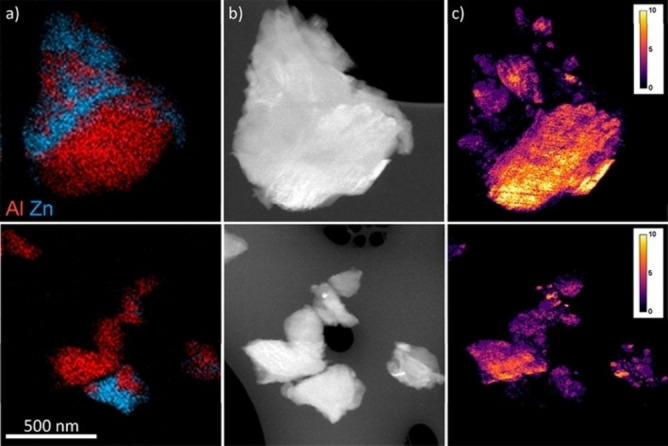
Scanning transmission electron microscopy of MOF‐CHGC particles of (MIL‐53)_0.6_(*a*_g_ZIF‐62)_0.4_ (upper) and (MIL‐53)_0.9_(*a*_g_ZIF‐62)_0.1_ (lower) samples. **a**. STEM‐EDS maps showing compositional maps of Al (red) and Zn (blue) metal centres. **b**. Annular dark‐field images. **c**. Crystallinity maps indicating the number of Bragg peaks as a function of probe position in SED data (adapted with permission, copyright 2019, American Chemical Society).^[47]^

General characterisation should also include microscopic techniques, such as scanning transmission electron microscopy (STEM). Energy dispersive spectroscopy (EDS) analysis and mapping should be carried out to investigate the presence of the different atomic elements contained within the blends and composites. Moreover, EDS mapping will provide insights into the homogeneous or heterogeneous elemental distribution of the blends and composites at the microscale, which is connected to the mechanical properties of the bulk composite (Figure 5). Additionally, STEM‐EDS tomography may give valuable information by reconstructing a complete 3D shard of the composites, especially when the MOF particles are smaller than 200 nm.^[41]^

However, the structural characterisation of highly disordered materials is challenging because amorphous materials are harder to characterise than their crystalline counterparts. Given the presence of only diffuse scattering in amorphous materials, atomic structures are not readily deduced. An X‐ray pair distribution function (PDF) measurement gives atomic distance information in real space by yielding atom‐atom correlation histograms, with histograms containing heavier elements contributing to the PDF more strongly. Moreover, small and overlapping contributions may be deciphered through the use of principal component analysis (PCA) and Non‐Negative Matrix Factorisation (NMF) performed on the PDF data^[53]^ As such, a complete characterisation using advanced synchrotron tools is critical to obtain information on the interfacial interactions between the glass matrix and the MOF. Following this line, molecular dynamics simulations may be extremely useful to study chemical compatibility at the molecular scale at the interface of both materials and their diffusion abilities.^[54]^

## Conclusions and Future Perspectives

In this article, we propose a new class of hybrid materials by combining the crystalline or glassy phase of MOFs, with inorganic glasses. The resultant materials are called MOF‐CIGCs, or blends. Seminal works have demonstrated the advantages of embedding MOF particles in a glass matrix to exploit the high surface areas of MOFs. The resulting composite exhibited increased CO_2_ uptake values compared with the starting materials.

In such cases, it is foreseen that the inorganic glass will flow around the MOF particles to create a heterogenous composite when heated to temperatures higher than the glass *T*_g_. In addition to creating composites with crystalline MOFs, inorganic glasses can also be combined with MOF glasses to create blends. By creating these new materials, the advantageous properties of MOFs can be exploited, bringing these materials closer to large‐scale industrial application by utilising the workability of glasses, many of which are already commercially significant. Moreover, an understanding of these novel composites and blends through advanced characterisation and bulk‐property testing will open exciting avenues in the field of materials which combine the best of both hybrid crystalline and glassy worlds.

## Conflict of interest

The authors declare no conflict of interest.

1

## Biographical Information

*Ashleigh M. Chester obtained her BSc degree in Chemistry from the University of Warwick in 2018 and completed her MSc at the University of St. Andrews in 2020. She is currently a Ph.D. student at the Department of Materials Science and Metallurgy (University of Cambridge), working on the development of novel hybrid materials comprising inorganic glasses and metal‐organic frameworks (MOFs)*.

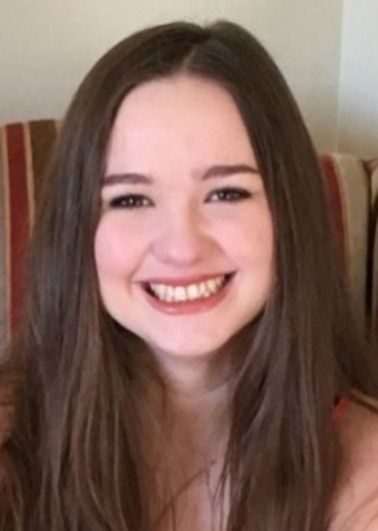

## Biographical Information

*Dr. Celia Castillo‐Blas obtained her Ph.D. degree in 2019 from the Institute of Materials Science of Madrid (ICMM–CSIC), Spain, where she focused on the design of metal‐organic frameworks (MOFs) with metal‐cation arrangement control in the secondary building units. After completing her Ph.D., she moved to Universidad Autónoma de Madrid (Spain) for a postdoctoral position working on the post‐synthetic modification of Zr‐MOFs. Currently, she is a postdoctoral research associate at the Department of Materials Science and Metallurgy (University of Cambridge) focused on the preparation and characterisation of MOFs‐inorganic glasses composites*.

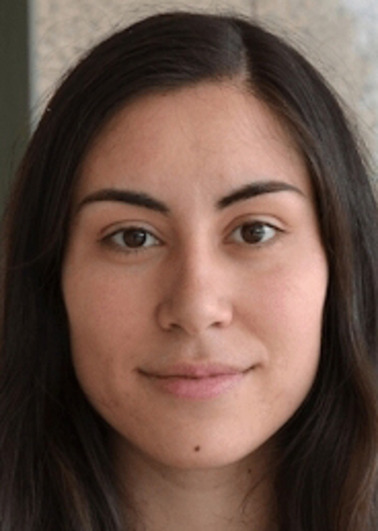

## Biographical Information

*Prof. Lothar Wondraczek obtained his doctorate in Materials Science from Clausthal University of Technology in 2003. Between 2005–2008 he was a Senior Research Scientist at Corning's European Technology Center (Avon, France) before moving to the University of Erlangen‐Nuremberg as a professor of Materials Science (2008–2012). He is currently the Chair of Glass Chemistry and has been the director of the Otto Schott Institute of Materials Research at the University of Jena since 2012. His work has been recognised with various national and international awards, including the Adolf‐Dietzel Industrial Award, the W. H. Zachariensen Award, and an ERC Consolidator grant*.

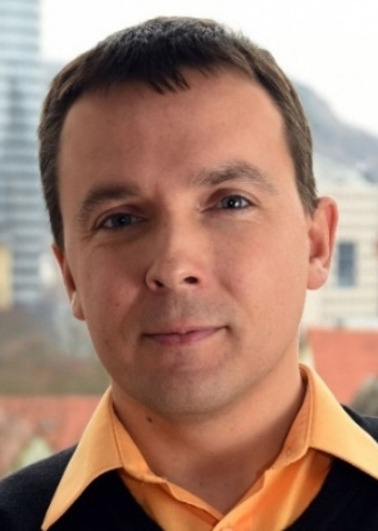

## Biographical Information

*Professor David A. Keen was awarded his Ph.D. at the University of Oxford in 1990, studying the structure of disordered materials by neutron scattering. He has worked on the local structure arrangements of condensed matter and has developed total scattering (pair distribution function, PDF) as well as experimental and reverse Monte Carlo computational methods to probe the relationship between structural disorder and the resulting physical properties of materials. He is currently a visiting professor at the Physics Department in Oxford University and research scientist at the ISIS Neutron Scattering Facility at the Rutherford Appleton Laboratory (Oxfordshire, UK)*.

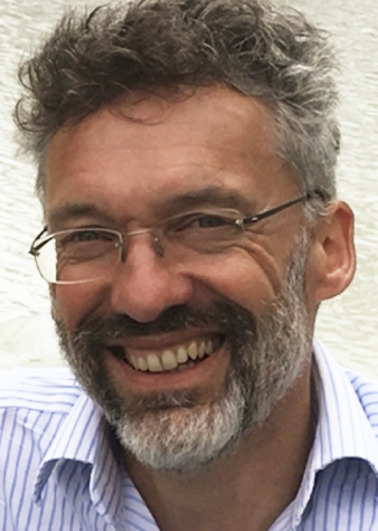

## Biographical Information

*Dr Thomas Bennett obtained his Ph.D. from the University of Cambridge in 2012, working under the supervision of Professor Anthony Cheetham FRS on the physical properties of hybrid frameworks. Currently, he is an Assistant Professor at the Department of Materials Science and Metallurgy (University of Cambridge), where his group focuses on hybrid melt‐quenched glasses, stimuli‐responsive framework behaviour and glass‐based composites. He is also currently vice‐chair of the international MOF advisory committee and has been a visiting researcher at the University of Canterbury New Zeeland |Te Whare Wānanga o Waitaha, University of Kyoto and the Wuhan University of Technology*.

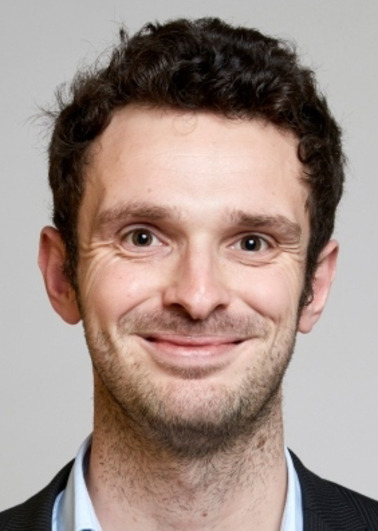

## Data Availability

Data sharing is not applicable to this article as no new data were created or analyzed in this study.
